# Método de búsqueda activa comunitaria para la captación de gestantes y puérperas en Ecuador

**DOI:** 10.26633/RPSP.2017.53

**Published:** 2017-05-15

**Authors:** Jakeline Calle Roldán, Cecilia Acuña, Paulina Ríos

**Affiliations:** 1 Subsecretaría de Gobernanza de la Salud Ministerio de Salud Pública Quito Ecuador Subsecretaría de Gobernanza de la Salud, Ministerio de Salud Pública, Quito, Ecuador.; 2 Organización Panamericana de la Salud Organización Panamericana de la Salud Washington, DC Estados Unidos de América Organización Panamericana de la Salud, Washington, DC, Estados Unidos de América.; 3 Universidad Central del Ecuador Universidad Central del Ecuador Quito Ecuador Universidad Central del Ecuador. Facultad de Ciencias Médicas, Quito, Ecuador.

**Keywords:** Embarazo, periodo postparto, participación comunitaria, planificación, servicios de salud comunitaria, Ecuador, Pregnancy, postpartum period, community participation, planning, community health services, Ecuador

## Abstract

**Objetivo.:**

Documentar y analizar la experiencia ecuatoriana en la aplicación del método de Búsqueda Activa Comunitaria (BAC) en la captación de mujeres gestantes y puérperas en Ecuador.

**Métodos.:**

Se realizó un estudio descriptivo transversal de la información obtenida de la aplicación de la BAC de mujeres gestantes y puérperas en las áreas de influencia geográfica de 200 establecimientos de atención primaria del Ministerio de Salud Pública de Ecuador.

**Resultados.:**

Se visitaron 460.451 casas en 20 provincias. Se identificaron 15.622 embarazadas y 4.014 puérperas. El 89% (13.875) de las embarazadas identificadas se había sometido al menos a un control prenatal y 70% (4 014) de las puérperas identificadas, al menos a un control posterior a su parto o cesárea. El 29% de las embarazadas (4 601) identificadas tenían riesgo potencial. Orellana y Sucumbíos fueron las provincias con menor porcentaje de embarazadas con al menos un control prenatal y con el menor porcentaje de puérperas con al menos un control postparto. Para esta actividad se conformó un total de 3 951 brigadas a nivel nacional.

**Conclusiones.:**

El método de BAC fue valioso para identificar embarazadas y puérperas que no habían sido captadas por el sistema de salud, y especialmente detectar su situación de riesgo, además de las ventajas del trabajo participativo en el proceso de captación, sobre todo con el apoyo de universidades con carreras vinculadas con la salud. Las bajas coberturas de control de puérperas señalan la importancia de conocer las causas de la falta de adherencia a estos controles. Se requiere sistematizar experiencias similares para introducir mejoras en el procedimiento.

## Abstract (en)

**Objective.:**

Document and analyze Ecuador’s experience using active community case-finding for uptake of pregnant and postpartum women in Ecuador.

**Methods.:**

A cross-sectional descriptive study was conducted of information obtained on active community case-finding of pregnant and postpartum women in the catchment areas of 200 primary care facilities of Ecuador’s Ministry of Public Health.

**Results.:**

Visits were made to 460 451 homes in 20 provinces; 15 622 pregnant women and 4 014 postpartum women were identified. Of the pregnant women, 89% (13 875) had had at least one prenatal check-up, while 70% of postpartum women (4 014) had had at least one post-delivery or post-caesarean check-up. Potential risk was identified in 29% of pregnant women (4 601). Orellana and Sucumbíos provinces had the lowest percentages of pregnant women with at least one prenatal check-up and the lowest percentages of postpartum women with at least one postpartum checkup. A total of 3 951 brigades were formed at the national level for this activity.

**Conclusions.:**

Active community case-finding was valuable in identifying pregnant and postpartum women who had not been captured by the health system, especially to detect their risk status, in addition to the advantages of participatory uptake, especially with support from universities with health majors. Low coverage for postpartum check-ups underscores the importance of learning why women do not receive this care. Similar experiences need to be systematized to improve the process.

Alrededor de 830 mujeres mueren cada día en el mundo por una causa relacionada con el embarazo, el parto o el postparto según datos de la Organización Mundial de la Salud (0MS) (1). Aunque en América Latina y el Caribe la mortalidad materna se redujo 50%, durante 2013 unas 9 300 mujeres perdieron su vida por estas causas. Ecuador ha contribuido notablemente a la reducción de la mortalidad materna (se redujo 65% entre 1990 y 2015). Sin embargo, en 2014 se registraron 166 muertes en el país, nueve más que en 2013 (2).

El control prenatal (CPN) es un conjunto de actividades y procedimientos que el equipo de salud ofrece a la embarazada con la finalidad de identificar factores de riesgo en la gestante y enfermedades que puedan afectar el curso normal del embarazo y la salud del recién nacido (3). La cobertura de atención prenatal se define como la proporción de mujeres que acudieron por lo menos una vez al control prenatal del total de partos (4), Estudios realizados en América Latina muestran que existe una relación inversa entre el CPN y la mortalidad materna y neonatal (5, 6) y que el riesgo de morir por una causa materna es de 2,1 a 3,2 veces más alta si la mujer no recibe controles prenatales (7, 8). Entre las causas de muerte materna clasificadas por demoras (9), la demora en tomar la decisión de buscar ayuda (demora 1), la demora relacionada con el retraso en acceder a un servicio de salud (demora 2) y la demora en recibir el tratamiento adecuado (demora 3) se podrían evitar mediante la captación temprana de la embarazada y un adecuado control prenatal.

En Ecuador, el CPN aumentó a 52% entre 1999 y 2004 a partir de la aplicación de la Ley de Maternidad Gratuita y Atención a la Infancia, promulgada en 1994 (10), y a 71,5%, entre 2007 y 2012, cuando la nueva Constitución de la República de 2008 estableció el acceso gratuito a los servicios de salud a todos los grupos de población. En 2009, la cobertura de al menos un control y de al menos 4 controles prenatales durante el embarazo alcanzaba 84 y 57%, respectivamente (10).

A pesar de estos avances y de la implementación de diversas estrategias para promover el control prenatal, aún no se alcanza la cobertura completa y persisten diferencias entre las áreas urbana y rural (81,4 y 73,7%, respectivamente) (11). Ello plantea la necesidad de desarrollar estrategias más eficientes de captación de las embarazadas (13-16).

La Búsqueda Activa Comunitaria (BAC) se define como una *“acción proactiva para la detección de casos que por cualquier razón no fueron notificados o ingresados al sistema. Es una fuente de información más y un instrumento de control de calidad de la vigilancia de rutina, porque permite detectar casos que escapan al sistema” *(12).

La BAC es un método ampliamente utilizado en vigilancia epidemiológica, especialmente para actividades de inmunización. En 2014 fue adoptado y adaptado por el Ministerio de Salud Pública de Ecuador (MSP) para la captación de mujeres gestantes y puérperas que no habían sido identificadas previamente y para hacer seguimiento nominal de aquellas que presentaran señales de peligro en su embarazo o periodo postparto, garantizando así su atención adecuada y oportuna (13, 14).

El objetivo de este artículo es documentar y analizar la experiencia ecuatoriana en la aplicación del método de BAC en la captación de mujeres gestantes y puérperas.

## MATERIALES Y MÉTODOS

Se realizó un estudio descriptivo transversal de la información obtenida con la BAC. Se consideraron todos los datos obtenidos de la captación, que se introdujeron en bases de datos con formato Excel. Se realizó un análisis descriptivo de cada una de las variables contenidas en el instrumento con el programa SPSS versión 19.

### Aplicación del método BAC

La primera fase consistió en el pilotaje. Se adaptaron y validaron los instrumentos y el procedimiento utilizados en los procesos de búsqueda activa comunitaria de casos de sarampión mediante un pilotaje en cuatro barrios de una parroquia rural de la ciudad de Quito, con la participación del personal del establecimiento de salud de la parroquia. En el pilotaje participaron 60 personas entre estudiantes universitarios de carreras de ciencias de la salud, líderes comunitarios y miembros del Gobierno Autónomo Descentralizado. En una jornada de seis horas se visitaron 1 539 casas en un área geográfica predefinida y en total se captaron 67 embarazadas y 4 puérperas.

Las definiciones operativas utilizadas fueron las siguientes. Casa efectiva (E), aquella donde habita una embarazada. Casa fallida (F), domicilio donde no se puede obtener información debido a que no se encuentra una persona mayor de edad que pueda proporcionar los datos. Casa no efectiva (NE), vivienda en la cual se ha realizado la entrevista corta a una persona mayor de edad y se ha verificado que no viven embarazadas. Embarazadas con control prenatal, mujeres grávidas a las cuales se ha realizado al menos un control prenatal en algún establecimiento de salud público o privado. Embarazadas sin control prenatal, aquellas que, al realizar la visita, no tienen el carné prenatal para verificar sus controles o las que declaran no recibir control prenatal. Embarazadas con potencial riesgo, gestantes con una o más de las siguientes características: sin controles prenatales, señales de peligro (sangrados, salida de líquido amniótico, convulsiones, dolor de cabeza, fiebre, entre otros) o morbilidad.

Las actividades realizadas durante el pilotaje incluyeron las siguientes: a) antes de la BAC, comunicados a las autoridades locales, socialización y organización del personal de salud, líderes comunitarios, actores locales y estudiantes de carreras relacionadas con las ciencias de la salud previamente sensibilizados y capacitados; b) mapeo e identificación de sectores para cada barrio, capacitación in situ sobre el método, utilización de herramientas de recolección, análisis y validación de la información recogida a todos los participantes; c) identificación y selección de un establecimiento de salud para la atención médica de los casos que lo requirieran; d) durante la BAC, distribución de las brigadas según los sectores definidos; e) visita puerta a puerta, registro de las embarazadas, referencia inmediata de aquellas con potencial riesgo, registro en la agenda de citas médicas en el establecimiento del MSP más cercano a las embarazadas y puérperas identificadas, análisis de los resultados y retroalimentación con líderes locales, y f) después de la BAC, reprogramación de visitas a casas fallidas, actualización de las herramientas de seguimiento comunitario (mapas parlantes, registros obstétricos, tarjeteros), programación de reuniones para análisis de la información, y planificación de intervenciones.

Posible riesgo se definió con los siguientes cuatro criterios: 1) no acudir a control prenatal; 2) edad de la paciente: todo embarazo de mujeres < 19 años y > 35 años se considera de riesgo; 3) antecedentes de enfermedades que signifiquen incremento de riesgo durante el embarazo y el parto, y 4) presencia actual de signos de alarma (dolor de cabeza, sangrado, salida de agua de fuente, fiebre, dificultad para respirar, nausea, vómitos abundantes.)

La segunda fase se consideró la aplicación de la BAC a escala nacional. En diciembre de 2015, se aplicó la BAC en las áreas de influencia geográfica de 200 establecimientos de atención primaria del MSP. La selección de las áreas geográficas se realizó según el porcentaje de captación de embarazadas de cada establecimiento de salud obtenido del “censo obstétrico” (la matriz utilizada por los establecimientos de salud para consolidar información sobre pacientes obstétricas y compartirla a escala nacional), los datos de cobertura de control prenatal del Registro Diario Automatizado de Consultas y Atenciones Ambulatorias (RDACAA) del MSP, y la información sobre los lugares de residencia donde se notificaron muertes maternas en el periodo 2013-2014. Los resultados que se describen a continuación corresponden a la información consolidada de los 200 establecimientos de salud.

**CUADRO 1:** Casas efectivas y no efectivas y embarazadas y puérperas identificadas por provincias, Ecuador

Provincia	Casas visitadas	Casas efectivas y no efectivas		Embarazadas identificadas		Mujeres en postparto identificadas	
		No.	%	No.	%	No.	%
Azuay	1 856	1 815	98	77	4,2	15	0,8
Bolivar	16 331	15 936	98	407	2,6	102	0,6
Cañar	1 946	1 594	82	43	2,7	11	0,7
Chimborazo	9 612	7 471	78	447	6,0	132	1,8
Cotopaxi	7 070	5 771	82	306	5,3	78	1,4
El oro	11 221	10 220	91	461	4,5	95	0,9
Esmeraldas	14 125	13 666	97	590	4,3	188	1,4
Guayas	131 394	117 721	90	4 582	3,9	1 181	1,0
Imbabura	2 013	1 971	98	61	3,1	15	0,8
Loja	7 432	6 860	92	258	3,8	52	0,8
Los ríos	69 404	65 219	94	2 943	4,5	664	1,0
Manabí	27 797	23 028	83	928	4,0	234	1,0
Morona Santiago	2 268	2 145	95	90	4,2	20	0,9
Orellana	5 592	4 103	73	262	6,4	58	1,4
Pastaza	2 629	2 602	99	80	3,1	19	0,7
Pichincha	82 580	75 148	91	2 007	2,7	700	0,9
Santa Elena	52 962	47 891	90	1 647	3,4	348	0,7
Sucumbios	5 751	4 950	86	313	6,3	73	1,5
Tungurahua	8 074	7 073	88	104	1,5	24	0,3
Zamora Chinchipe	394	381	97	16	4,2	5	1,3
Total	460 451	415 565	90	15 622		4 014	

*Fuente:* Informes de Coordinaciones Zonales de Salud. Elaborado por la Gerencia Institucional de Disminución de Mortalidad Materna, 2015.

**CUADRO 2:** Total de embarazadas identificadas, con control prenatal y sin control prenatal, por zonas, Ecuador

Provincia	Embarazadas identificadas	Gestantes con al menos un control prenatal		Mujeres en postparto con al menos un control	
		No.	%	No.	%
Orellana	262	204	78	29	50
Sucumbios	313	248	79	40	55
Manabí	928	760	82	143	61
Bolivar	407	337	83	70	69
Esmeraldas	590	490	83	152	81
Azuay	77	66	86	5	33
Los ríos	2 943	2 553	87	425	64
Santa Elena	1 647	1 481	90	295	85
Guayas	4 582	4 117	90	750	64
Pastaza	80	73	91	11	58
Loja	258	236	91	30	58
Imbabura	61	56	92	11	73
Pichincha	2 007	1 855	92	567	81
Cañar	43	40	93	6	55
Morona Santiago	90	84	93	14	70
El oro	461	427	93	78	82
Tungurahua	104	98	94	15	63
Chimborazo	447	434	97	94	71
Cotopaxi	306	300	98	56	72
Zamora Chinchipe	16	16	100	5	10
Total	15 622	13 875	89	2 796	7

*Fuente:* Informes de Coordinaciones Zonales de Salud. Elaborado por la Gerencia Institucional de Disminución de Mortalidad Materna, 2015.

## RESULTADOS

En total, se visitaron 460.451 casas en 20 provincias. De ellas, 90% se consideraron efectivas o no efectivas, y 10%, fallidas (cuadro 1). Se identificaron 15.622 embarazadas y 4 014 puérperas.

El 89% (13.875) de las embarazadas identificadas se había sometido al menos un control prenatal y 70% (4 014) de las puérperas identificadas, al menos a un control posterior a su parto o cesárea. Las provincias con menor porcentaje de embarazadas con al menos un control prenatal y con el menor porcentaje de puérperas con al menos un control postparto fueron Orellana y Sucumbíos. En la provincia de Azuay se registró un porcentaje notoriamente bajo de puérperas: al menos un control postnatal (33%) (cuadro 2). El 29% de las embarazadas (4 601) identificadas tenían potencial riesgo. Las provincias que más embarazadas notificaron con potencial riesgo fueron Bolívar, Cotopaxi y Tungurahua, todas ellas con porcentajes por encima de 50% (cuadro 3).

**CUADRO 3:** Total de embarazadas identificadas y porcentaje con potencial riesgo, Ecuador

Provincia	Embarazadas identificadas	Gestantes con potencial riesgo	
	No.	No.	%
Bolivar	407	226	56
Cotopaxi	306	158	52
Tungurahua	104	54	52
Pastaza	80	40	50
Zamora Chinchipe	16	8	50
Morona Santiago	90	38	42
Guayas	4 582	1 854	40
Imbabura	61	22	36
Los ríos	2 943	1 031	35
Santa Elena	1 647	471	29
Esmeraldas	590	150	25
Orellana	262	65	25
Loja	258	54	21
Sucumbios	313	61	19
Manabí	928	167	18
El oro	461	76	16
Chimborazo	447	51	11
Pichincha	2 007	75	4
Azuay	77	0	0
Cañar	43	0	0
Total	15 622	4 601	29

*Fuente:* Informes de Coordinaciones Zonales de Salud. Elaborado por la Gerencia Institucional de Disminución de Mortalidad Materna, 2015.

En la aplicación de la BAC participaron 1 367 estudiantes universitarios, 560 comités locales de salud, 400 técnicos en atención primaria en salud (TAPS), 157 funcionarios de los Gobiernos Autónomos Descentralizados (GAD) y 5 411 trabajadores de salud, que conformaron un total de 3 951 brigadas a nivel nacional. Las provincias con menor porcentaje de casas fallidas fueron Pastaza, Azuay, Bolívar e Imbabura (cuadro 4).

**CUADRO 4:** Personal que participó en las brigadas, Ecuador

Provincia	Personal de salud	Estudiantes universitarios	Comités Locales de Salud	Técnicos de atención priemaria de salud	Gobiernos autónomos descentralizados	Otros	Brigadas	Casas visitadas
	No.	No.	No.	No.	No.	No.	No.	No.
Guayas	1 345	80	249	47	90	162	922	131 394
Pichincha	822	301	14	154	0	126	521	82 580
Los ríos	653	139	95	53	21	95	511	69 404
Bolivar	256	28	50	17	10	10	429	16 331
Santa Elena	647	0	15	11	2	1	315	52 962
Manabí	146	248	8	16	0	0	304	27 797
El oro	172	86o	17	14	11	98	133	11 221
Cañar	82		11	2		13	108	1 946
Azuay	107						108	1 856
Esmeraldas	343	61	32	28	15	20	99	14 125
Tungurahua	64	338	2	3	2		96	8 074
Cotopaxi	61	73	9	25	3	2	79	7 070
Chimborazo	147	13	10	15	0	213	78	9 612
Sucumbios	71	0	10	9	0	87	74	5 751
Imbabura	86	0	1	0	1	0	59	2 013
Loja	257	0	30	6	0	13	48	7 432
Pastaza	52	0	0	0	0	0	26	2 629
Morona Santiago	25		1				26	2 268
Orellana	68	0	0	0	2	23	11	5 592
Zamora Chinchipe	7	0	6	0	0	4	4	394
Total	5 411	1 367	560	400	157	867	3 951	460 451

*Fuente:* Informes de Coordinaciones Zonales de Salud. Elaborado por la Gerencia Institucional de Disminución de Mortalidad Materna, 2015.

## DISCUSIÓN

El porcentaje de embarazadas con al menos un control prenatal de 89% detectado indica un aumento en relación con años anteriores (15). Sin embargo, esta cobertura es menor que la notificada por Colombia (97%) (16) o Perú (98,4%) (17). La baja captación de embarazadas y puérperas observada en las provincias de Orellana y Sucumbíos concuerda con determinantes sociales de ambas provincias relacionados con la pobreza (Necesidades Básicas Insatisfechas 62,9 y 59,4%, respectivamente) (18) y barreras geográficas y culturales de acceso a los servicios de salud. Aunque en promedio la cobertura de al menos un control postnatal en puérperas fue 70%, las bajas coberturas observadas en Orellana y Azuay señalan la importancia que tiene conocer las causas de la falta de adherencia a estos controles.

Asimismo, llama la atención que 29% de las gestantes captadas con este método se encontraban con potencial riesgo. Ello sugiere que podría existir un problema de calidad de los controles prenatales, lo cual se ha observado también en otros países y constituye un factor que debe tenerse en cuenta además de la cobertura (19).

El método de BAC fue valioso para identificar embarazadas y puérperas que no habían sido captadas por el sistema de salud y, en particular, para detectar su situación de riesgo. Se comprobó, además, que la estimación inicial de la población de embarazadas era menor que la observada. Ello subraya la necesidad de conocer con mayor exactitud a la población en el área geográfica de influencia mediante estrategias tales como la adscripción territorial, lo que permitiría al equipo de salud asumir la responsabilidad del cuidado integral de la población que tiene a su cargo y mejorar la planificación de actividades (20).

Entre las limitaciones de este estudio cabe destacar que la valoración del riesgo fue cualitativa, que la herramienta no contempla una valoración cuantitativa y no permite identificar si las embarazadas identificadas eran beneficiarias del subsistema público, privado o de la Seguridad Social.

El estudio pone de manifiesto que es posible explorar y adaptar herramientas del campo de salud pública a la captación de embarazadas y puérperas. A partir de esta experiencia, la BAC se ha utilizado para mejorar la identificación y el seguimiento de embarazadas y puérperas, mediante la búsqueda cotidiana en establecimientos de salud del MSP. La aplicación exitosa de esta herramienta por el personal del primer nivel de atención en colaboración con estudiantes universitarios y líderes comunitarios mostró las ventajas del trabajo participativo en el proceso de captación, especialmente con el apoyo de universidades con carreras vinculadas con la salud. La marcada participación de diversos sectores de la sociedad civil sugiere que se puede seguir contando con ellos para la aplicación rutinaria del método.

Aunque existen pocas experiencias de aplicación de la BAC para captar embarazadas y puérperas en otros países, se sugiere sistematizar experiencias similares para introducir mejoras en el procedimiento. Para los países interesados en aplicar la herramienta debe subrayarse la utilidad de marcarse metas geográficas de cobertura (total de viviendas visitadas por sectores), incluir variables para medir la cobertura y la concentración (el número de controles prenatales), y emplear la herramienta en países donde las coberturas de CPN son bajas o en localidades geográficas de difícil acceso.

Se recomienda que los procesos de captación activa sean integrales y se coordinen con otras estrategias, como la búsqueda por rumores o la búsqueda incidental en las visitas por otras actividades, en el marco de la “Vigilancia Comunitaria” (21), que es complementaria a la captación pasiva actual. Por último, se recomienda, asimismo, optimizar las actividades del personal de salud apoyando a los agentes comunitarios, ya que pueden proporcionar información más realista sobre los lugares, días u horas para realizar actividades de búsqueda activa. Ello permitiría, además, mejorar la participación y el compromiso de toda la comunidad, así como realizar un seguimiento constante de las embarazadas y puérperas, lo cual podría contribuir así a la reducción de mortalidad materna.

### Financiación.

Esta actividad fue financiada por cada una de las Coordinaciones Zonales y sus direcciones distritales, que pertenecen al Ministerio de Salud, sin embargo algunos aspectos logísticos fueron solventados voluntariamente por los Gobiernos Locales.

### Agradecimientos.

Los autores agradecen a los analistas de la Gerencia de Implementación de Reducción de Muerte Materna que apoyaron este trabajo en campo, a los equipos de la Dirección Distrital 17D09, del Establecimiento de salud de Tumbaco, de la zona de salud No. 9., a docentes y estudiantes de la Carrera de Obstetricia, Facultad de Ciencias Médicas, Universidad Central del Ecuador, y a los docentes y estudiantes de la Carrera de Enfermería, Universidad Católica del Ecuador, grupo de colaboradoras del GAD parroquial, su apoyo en el proceso de pilotaje. La experiencia paralela de los profesionales de la Dirección Distrital Chimbo, Zona 3, contribuyó a enriquecer la implantación del método a escala nacional. Asimismo, expresan su agradecimiento a los profesionales que de manera incansable apoyaron en el proceso de búsqueda y captación en las nueve zonas de Ecuador y a todas las personas que colaboraron en este esfuerzo en cada uno de sus territorios de influencia.

### Declaración.

Las opiniones expresadas en este manuscrito son responsabilidad del autor y no reflejan necesariamente los criterios ni la política de la *RPSP/ PAJPH y*/o de la OPS.
